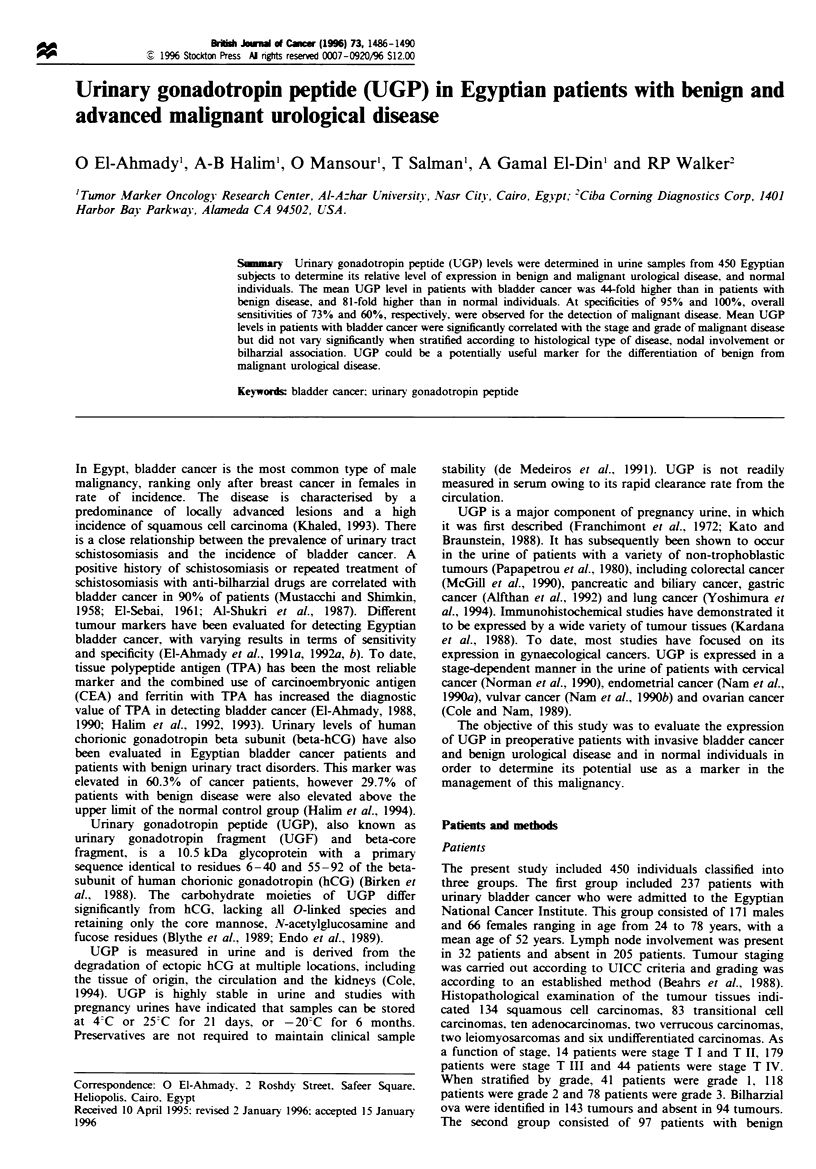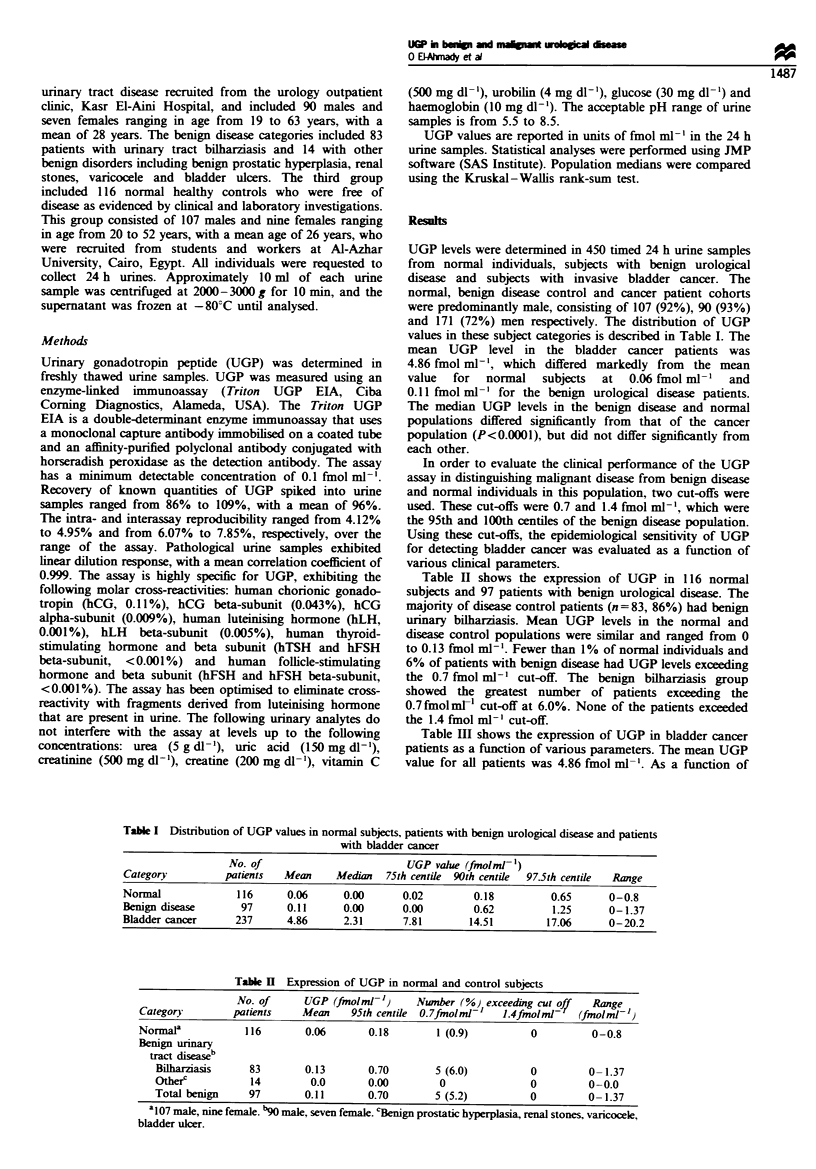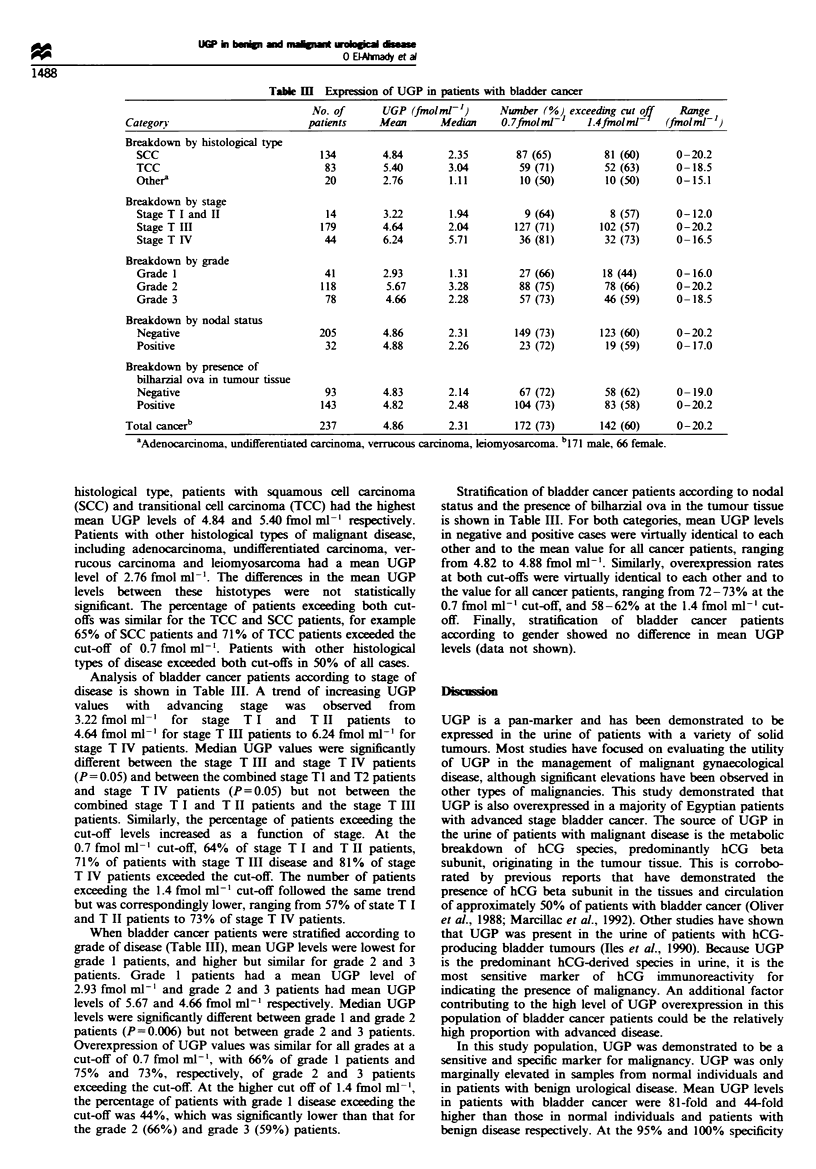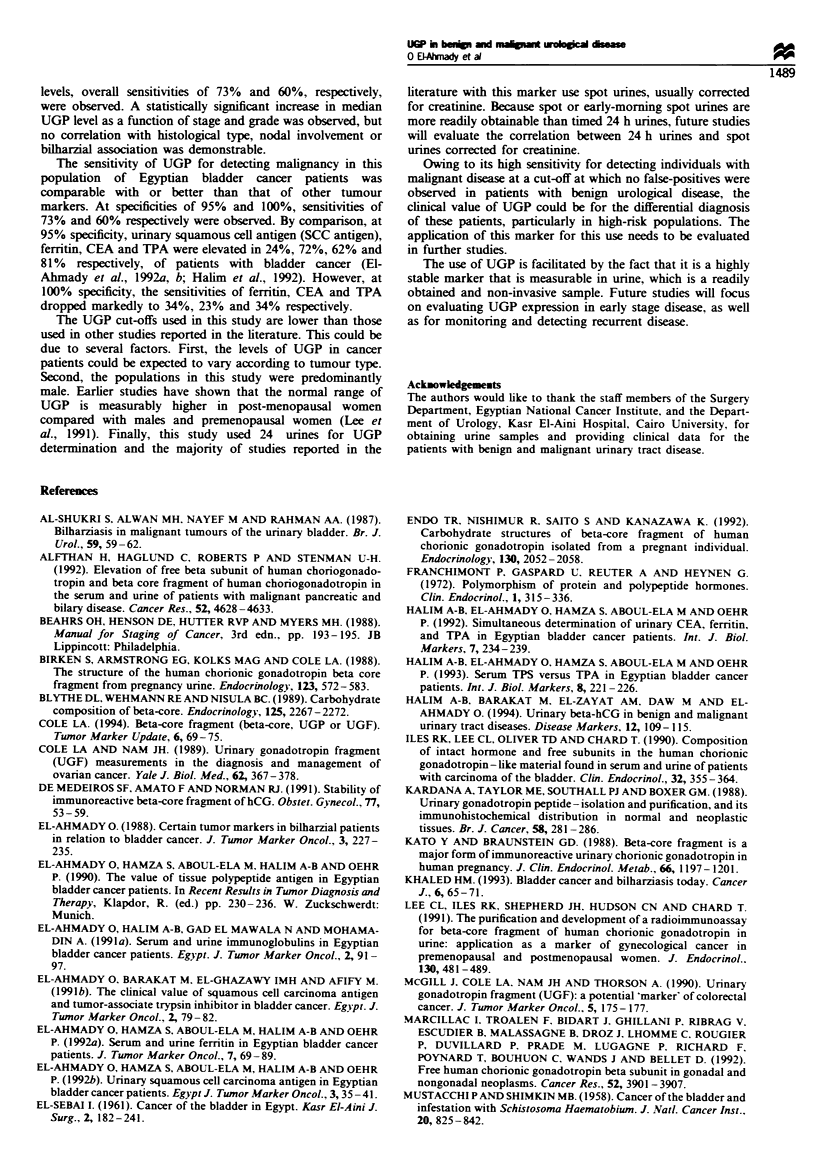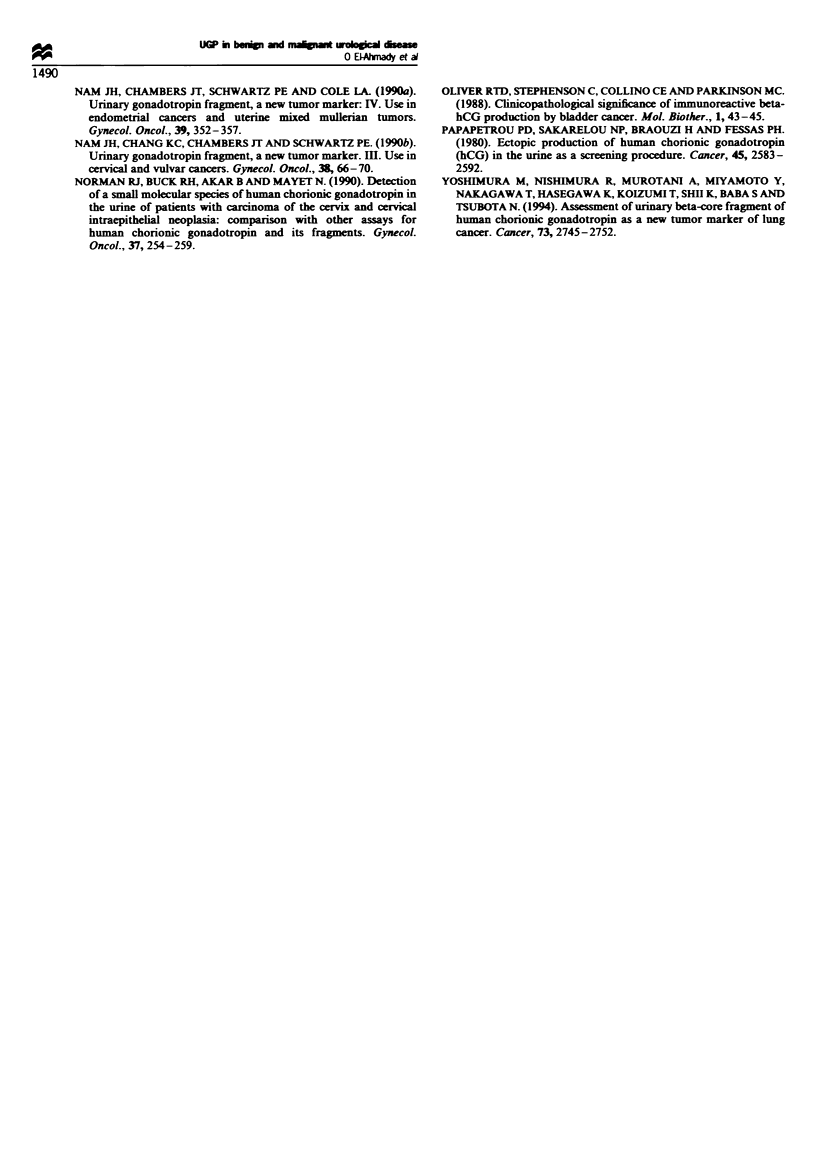# Urinary gonadotropin peptide (UGP) in Egyptian patients with benign and advanced malignant urological disease.

**DOI:** 10.1038/bjc.1996.281

**Published:** 1996-06

**Authors:** O. el-Ahmady, A. B. Halim, O. Mansour, T. Salman, A. G. el-Din, R. P. Walker

**Affiliations:** Tumor Marker Oncology Research Center, Al-Azhar University, Cairo, Egypt.

## Abstract

Urinary gonadotropin peptide (UGP) levels were determined in urine samples from 450 Egyptian subjects to determine its relative level of expression in benign and malignant urological disease, and normal individuals. The mean UGP level in patients with bladder cancer was 44-fold higher than in patients with benign disease, and 81-fold higher than in normal individuals. At specificities of 95% and 100%, overall sensitivities of 73% and 60%, respectively, were observed for the detection of malignant disease. Mean UGP levels in patients with bladder cancer were significantly correlated with the stage and grade of malignant disease but did not vary significantly when stratified according to histological type of disease, nodal involvement or bilharzial association. UGP could be a potentially useful marker for the differentiation of benign from malignant urological disease.


British Journal of Cancer (1996) 73, 1486-1490

© 1996 Stockton Press All rights reserved 0007-0920/96 $12.00

### Urinary gonadotropin peptide (UGP) in Egyptian patients with benign and advanced malignant urological disease

O El-Ahmady[1], A-B Halim[1], O Mansour[1], T Salman[1], A Gamal El-Din[1] and RP Walker[2]

[1]*Tumor Marker Oncology Research Center, Al-Azhar University, Nasr City, Cairo, Egypt;* [2]*Ciba Corning Diagnostics Corp, 1401 Harbor Bay Parkway, Alameda CA 94502, USA.*

**Summary** Urinary gonadotropin peptide (UGP) levels were determined in urine samples from 450 Egyptian subjects to determine its relative level of expression in benign and malignant urological disease, and normal individuals. The mean UGP level in patients with bladder cancer was 44-fold higher than in patients with benign disease, and 81-fold higher than in normal individuals. At specificities of 95% and 100%, overall sensitivities of 73% and 60%, respectively, were observed for the detection of malignant disease. Mean UGP levels in patients with bladder cancer were significantly correlated with the stage and grade of malignant disease but did not vary significantly when stratified according to histological type of disease, nodal involvement or bilharzial association. UGP could be a potentially useful marker for the differentiation of benign from malignant urological disease.

**Keywords:** bladder cancer; urinary gonadotropin peptide

In Egypt, bladder cancer is the most common type of male malignancy, ranking only after breast cancer in females in rate of incidence. The disease is characterised by a predominance of locally advanced lesions and a high incidence of squamous cell carcinoma (Khaled, 1993). There is a close relationship between the prevalence of urinary tract schistosomiasis and the incidence of bladder cancer. A positive history of schistosomiasis or repeated treatment of schistosomiasis with anti-bilharzial drugs are correlated with bladder cancer in 90% of patients (Mustacchi and Shimkin, 1958; El-Sebai, 1961; Al-Shukri *et al.*, 1987). Different tumour markers have been evaluated for detecting Egyptian bladder cancer, with varying results in terms of sensitivity and specificity (El-Ahmady *et al.*, 1991*a*, 1992*a*, *b*). To date, tissue polypeptide antigen (TPA) has been the most reliable marker and the combined use of carcinoembryonic antigen (CEA) and ferritin with TPA has increased the diagnostic value of TPA in detecting bladder cancer (El-Ahmady, 1988, 1990; Halim *et al.*, 1992, 1993). Urinary levels of human chorionic gonadotropin beta subunit (beta-hCG) have also been evaluated in Egyptian bladder cancer patients and patients with benign urinary tract disorders. This marker was elevated in 60.3% of cancer patients, however 29.7% of patients with benign disease were also elevated above the upper limit of the normal control group (Halim *et al.*, 1994).

Urinary gonadotropin peptide (UGP), also known as urinary gonadotropin fragment (UGF) and beta-core fragment, is a 10.5 kDa glycoprotein with a primary sequence identical to residues 6-40 and 55-92 of the beta-subunit of human chorionic gonadotropin (hCG) (Birken *et al.*, 1988). The carbohydrate moieties of UGP differ significantly from hCG, lacking all *O*-linked species and retaining only the core mannose, *N*-acetylglucosamine and fucose residues (Blythe *et al.*, 1989; Endo *et al.*, 1989).

UGP is measured in urine and is derived from the degradation of ectopic hCG at multiple locations, including the tissue of origin, the circulation and the kidneys (Cole, 1994). UGP is highly stable in urine and studies with pregnancy urines have indicated that samples can be stored at 4°C or 25°C for 21 days, or −20°C for 6 months. Preservatives are not required to maintain clinical sample stability (de Medeiros *et al.*, 1991). UGP is not readily measured in serum owing to its rapid clearance rate from the circulation.

UGP is a major component of pregnancy urine, in which it was first described (Franchimont *et al.*, 1972; Kato and Braunstein, 1988). It has subsequently been shown to occur in the urine of patients with a variety of non-trophoblastic tumours (Papapetrou *et al.*, 1980), including colorectal cancer (McGill *et al.*, 1990), pancreatic and biliary cancer, gastric cancer (Alfthan *et al.*, 1992) and lung cancer (Yoshimura *et al.*, 1994). Immunohistochemical studies have demonstrated it to be expressed by a wide variety of tumour tissues (Kardana *et al.*, 1988). To date, most studies have focused on its expression in gynaecological cancers. UGP is expressed in a stage-dependent manner in the urine of patients with cervical cancer (Norman *et al.*, 1990), endometrial cancer (Nam *et al.*, 1990*a*), vulvar cancer (Nam *et al.*, 1990*b*) and ovarian cancer (Cole and Nam, 1989).

The objective of this study was to evaluate the expression of UGP in preoperative patients with invasive bladder cancer and benign urological disease and in normal individuals in order to determine its potential use as a marker in the management of this malignancy.

### Patients and methods

### Patients

The present study included 450 individuals classified into three groups. The first group included 237 patients with urinary bladder cancer who were admitted to the Egyptian National Cancer Institute. This group consisted of 171 males and 66 females ranging in age from 24 to 78 years, with a mean age of 52 years. Lymph node involvement was present in 32 patients and absent in 205 patients. Tumour staging was carried out according to UICC criteria and grading was according to an established method (Beahrs *et al.*, 1988). Histopathological examination of the tumour tissues indicated 134 squamous cell carcinomas, 83 transitional cell carcinomas, ten adenocarcinomas, two verrucous carcinomas, two leiomyosarcomas and six undifferentiated carcinomas. As a function of stage, 14 patients were stage T I and T II, 179 patients were stage T III and 44 patients were stage T IV. When stratified by grade, 41 patients were grade 1, 118 patients were grade 2 and 78 patients were grade 3. Bilharzial ova were identified in 143 tumours and absent in 94 tumours. The second group consisted of 97 patients with benign

Correspondence: O El-Ahmady, 2 Roshdy Street, Safeer Square, Heliopolis, Cairo, Egypt

Received 10 April 1995; revised 2 January 1996; accepted 15 January 1996

urinary tract disease recruited from the urology outpatient clinic, Kasr El-Aini Hospital, and included 90 males and seven females ranging in age from 19 to 63 years, with a mean of 28 years. The benign disease categories included 83 patients with urinary tract bilharziasis and 14 with other benign disorders including benign prostatic hyperplasia, renal stones, varicocele and bladder ulcers. The third group included 116 normal healthy controls who were free of disease as evidenced by clinical and laboratory investigations. This group consisted of 107 males and nine females ranging in age from 20 to 52 years, with a mean age of 26 years, who were recruited from students and workers at Al-Azhar University, Cairo, Egypt. All individuals were requested to collect 24 h urines. Approximately 10 ml of each urine sample was centrifuged at 2000–3000 *g* for 10 min, and the supernatant was frozen at −80°C until analysed.

### *Methods*

Urinary gonadotropin peptide (UGP) was determined in freshly thawed urine samples. UGP was measured using an enzyme-linked immunoassay (*Triton* UGP EIA, Ciba Corning Diagnostics, Alameda, USA). The *Triton* UGP EIA is a double-determinant enzyme immunoassay that uses a monoclonal capture antibody immobilised on a coated tube and an affinity-purified polyclonal antibody conjugated with horseradish peroxidase as the detection antibody. The assay has a minimum detectable concentration of 0.1 fmol $ml^{-1}$. Recovery of known quantities of UGP spiked into urine samples ranged from 86% to 109%, with a mean of 96%. The intra- and interassay reproducibility ranged from 4.12% to 4.95% and from 6.07% to 7.85%, respectively, over the range of the assay. Pathological urine samples exhibited linear dilution response, with a mean correlation coefficient of 0.999. The assay is highly specific for UGP, exhibiting the following molar cross-reactivities: human chorionic gonadotropin (hCG, 0.11%), hCG beta-subunit (0.043%), hCG alpha-subunit (0.009%), human luteinising hormone (hLH, 0.001%), hLH beta-subunit (0.005%), human thyroid-stimulating hormone and beta subunit (hTSH and hFSH beta-subunit, <0.001%) and human follicle-stimulating hormone and beta subunit (hFSH and hFSH beta-subunit, <0.001%). The assay has been optimised to eliminate cross-reactivity with fragments derived from luteinising hormone that are present in urine. The following urinary analytes do not interfere with the assay at levels up to the following concentrations: urea (5 g $dl^{-1}$), uric acid (150 mg $dl^{-1}$), creatinine (500 mg $dl^{-1}$), creatine (200 mg $dl^{-1}$), vitamin C (500 mg $dl^{-1}$), urobilin (4 mg $dl^{-1}$), glucose (30 mg $dl^{-1}$) and haemoglobin (10 mg $dl^{-1}$). The acceptable pH range of urine samples is from 5.5 to 8.5.

UGP values are reported in units of fmol $ml^{-1}$ in the 24 h urine samples. Statistical analyses were performed using JMP software (SAS Institute). Population medians were compared using the Kruskal–Wallis rank-sum test.

### Results

UGP levels were determined in 450 timed 24 h urine samples from normal individuals, subjects with benign urological disease and subjects with invasive bladder cancer. The normal, benign disease control and cancer patient cohorts were predominantly male, consisting of 107 (92%), 90 (93%) and 171 (72%) men respectively. The distribution of UGP values in these subject categories is described in Table I. The mean UGP level in the bladder cancer patients was 4.86 fmol $ml^{-1}$, which differed markedly from the mean value for normal subjects at 0.06 fmol $ml^{-1}$ and 0.11 fmol $ml^{-1}$ for the benign urological disease patients. The median UGP levels in the benign disease and normal populations differed significantly from that of the cancer population ($P<0.0001$), but did not differ significantly from each other.

In order to evaluate the clinical performance of the UGP assay in distinguishing malignant disease from benign disease and normal individuals in this population, two cut-offs were used. These cut-offs were 0.7 and 1.4 fmol $ml^{-1}$, which were the 95th and 100th centiles of the benign disease population. Using these cut-offs, the epidemiological sensitivity of UGP for detecting bladder cancer was evaluated as a function of various clinical parameters.

Table II shows the expression of UGP in 116 normal subjects and 97 patients with benign urological disease. The majority of disease control patients ($n=83$, 86%) had benign urinary bilharziasis. Mean UGP levels in the normal and disease control populations were similar and ranged from 0 to 0.13 fmol $ml^{-1}$. Fewer than 1% of normal individuals and 6% of patients with benign disease had UGP levels exceeding the 0.7 fmol $ml^{-1}$ cut-off. The benign bilharziasis group showed the greatest number of patients exceeding the 0.7 fmol $ml^{-1}$ cut-off at 6.0%. None of the patients exceeded the 1.4 fmol $ml^{-1}$ cut-off.

Table III shows the expression of UGP in bladder cancer patients as a function of various parameters. The mean UGP value for all patients was 4.86 fmol $ml^{-1}$. As a function of

**Table I** Distribution of UGP values in normal subjects, patients with benign urological disease and patients with bladder cancer

| *Category* | *No. of patients* | *Mean* | *Median* | *75th centile* | *90th centile* | *97.5th centile* | *Range* |
|---|---|---|---|---|---|---|---|
| | | *UGP value (fmol $ml^{-1}$)* | | | | | |
| Normal | 116 | 0.06 | 0.00 | 0.02 | 0.18 | 0.65 | 0–0.8 |
| Benign disease | 97 | 0.11 | 0.00 | 0.00 | 0.62 | 1.25 | 0–1.37 |
| Bladder cancer | 237 | 4.86 | 2.31 | 7.81 | 14.51 | 17.06 | 0–20.2 |

**Table II** Expression of UGP in normal and control subjects

| *Category* | *No. of patients* | *UGP (fmol $ml^{-1}$) Mean* | *UGP (fmol $ml^{-1}$) 95th centile* | *Number (%) exceeding cut off 0.7 fmol $ml^{-1}$* | *Number (%) exceeding cut off 1.4 fmol $ml^{-1}$* | *Range (fmol $ml^{-1}$)* |
|---|---|---|---|---|---|---|
| Normal[a] | 116 | 0.06 | 0.18 | 1 (0.9) | 0 | 0–0.8 |
| Benign urinary tract disease[b] | | | | | | |
| Bilharziasis | 83 | 0.13 | 0.70 | 5 (6.0) | 0 | 0–1.37 |
| Other[c] | 14 | 0.0 | 0.00 | 0 | 0 | 0–0.0 |
| Total benign | 97 | 0.11 | 0.70 | 5 (5.2) | 0 | 0–1.37 |

[a]107 male, nine female. [b]90 male, seven female. [c]Benign prostatic hyperplasia, renal stones, varicocele, bladder ulcer.

Table III Expression of UGP in patients with bladder cancer

| Category | No. of patients | UGP ($fmol\,ml^{-1}$) Mean | Median | Number (%) exceeding cut off $0.7 fmol\,ml^{-1}$ | $1.4 fmol\,ml^{-1}$ | Range ($fmol\,ml^{-1}$) |
|---|---|---|---|---|---|---|
| Breakdown by histological type | | | | | | |
| SCC | 134 | 4.84 | 2.35 | 87 (65) | 81 (60) | 0-20.2 |
| TCC | 83 | 5.40 | 3.04 | 59 (71) | 52 (63) | 0-18.5 |
| Other[a] | 20 | 2.76 | 1.11 | 10 (50) | 10 (50) | 0-15.1 |
| Breakdown by stage | | | | | | |
| Stage T I and II | 14 | 3.22 | 1.94 | 9 (64) | 8 (57) | 0-12.0 |
| Stage T III | 179 | 4.64 | 2.04 | 127 (71) | 102 (57) | 0-20.2 |
| Stage T IV | 44 | 6.24 | 5.71 | 36 (81) | 32 (73) | 0-16.5 |
| Breakdown by grade | | | | | | |
| Grade 1 | 41 | 2.93 | 1.31 | 27 (66) | 18 (44) | 0-16.0 |
| Grade 2 | 118 | 5.67 | 3.28 | 88 (75) | 78 (66) | 0-20.2 |
| Grade 3 | 78 | 4.66 | 2.28 | 57 (73) | 46 (59) | 0-18.5 |
| Breakdown by nodal status | | | | | | |
| Negative | 205 | 4.86 | 2.31 | 149 (73) | 123 (60) | 0-20.2 |
| Positive | 32 | 4.88 | 2.26 | 23 (72) | 19 (59) | 0-17.0 |
| Breakdown by presence of bilharzial ova in tumour tissue | | | | | | |
| Negative | 93 | 4.83 | 2.14 | 67 (72) | 58 (62) | 0-19.0 |
| Positive | 143 | 4.82 | 2.48 | 104 (73) | 83 (58) | 0-20.2 |
| Total cancer[b] | 237 | 4.86 | 2.31 | 172 (73) | 142 (60) | 0-20.2 |

[a]Adenocarcinoma, undifferentiated carcinoma, verrucous carcinoma, leiomyosarcoma. [b]171 male, 66 female.

histological type, patients with squamous cell carcinoma (SCC) and transitional cell carcinoma (TCC) had the highest mean UGP levels of 4.84 and 5.40 $fmol\,ml^{-1}$ respectively. Patients with other histological types of malignant disease, including adenocarcinoma, undifferentiated carcinoma, verrucous carcinoma and leiomyosarcoma had a mean UGP level of 2.76 $fmol\,ml^{-1}$. The differences in the mean UGP levels between these histotypes were not statistically significant. The percentage of patients exceeding both cut-offs was similar for the TCC and SCC patients, for example 65% of SCC patients and 71% of TCC patients exceeded the cut-off of 0.7 $fmol\,ml^{-1}$. Patients with other histological types of disease exceeded both cut-offs in 50% of all cases.

Analysis of bladder cancer patients according to stage of disease is shown in Table III. A trend of increasing UGP values with advancing stage was observed from 3.22 $fmol\,ml^{-1}$ for stage T I and T II patients to 4.64 $fmol\,ml^{-1}$ for stage T III patients to 6.24 $fmol\,ml^{-1}$ for stage T IV patients. Median UGP values were significantly different between the stage T III and stage T IV patients ($P = 0.05$) and between the combined stage T1 and T2 patients and stage T IV patients ($P = 0.05$) but not between the combined stage T I and T II patients and the stage T III patients. Similarly, the percentage of patients exceeding the cut-off levels increased as a function of stage. At the 0.7 $fmol\,ml^{-1}$ cut-off, 64% of stage T I and T II patients, 71% of patients with stage T III disease and 81% of stage T IV patients exceeded the cut-off. The number of patients exceeding the 1.4 $fmol\,ml^{-1}$ cut-off followed the same trend but was correspondingly lower, ranging from 57% of state T I and T II patients to 73% of stage T IV patients.

When bladder cancer patients were stratified according to grade of disease (Table III), mean UGP levels were lowest for grade 1 patients, and higher but similar for grade 2 and 3 patients. Grade 1 patients had a mean UGP level of 2.93 $fmol\,ml^{-1}$ and grade 2 and 3 patients had mean UGP levels of 5.67 and 4.66 $fmol\,ml^{-1}$ respectively. Median UGP levels were significantly different between grade 1 and grade 2 patients ($P = 0.006$) but not between grade 2 and 3 patients. Overexpression of UGP values was similar for all grades at a cut-off of 0.7 $fmol\,ml^{-1}$, with 66% of grade 1 patients and 75% and 73%, respectively, of grade 2 and 3 patients exceeding the cut-off. At the higher cut off of 1.4 $fmol\,ml^{-1}$, the percentage of patients with grade 1 disease exceeding the cut-off was 44%, which was significantly lower than that for the grade 2 (66%) and grade 3 (59%) patients.

Stratification of bladder cancer patients according to nodal status and the presence of bilharzial ova in the tumour tissue is shown in Table III. For both categories, mean UGP levels in negative and positive cases were virtually identical to each other and to the mean value for all cancer patients, ranging from 4.82 to 4.88 $fmol\,ml^{-1}$. Similarly, overexpression rates at both cut-offs were virtually identical to each other and to the value for all cancer patients, ranging from 72-73% at the 0.7 $fmol\,ml^{-1}$ cut-off, and 58-62% at the 1.4 $fmol\,ml^{-1}$ cut-off. Finally, stratification of bladder cancer patients according to gender showed no difference in mean UGP levels (data not shown).

### Discussion

UGP is a pan-marker and has been demonstrated to be expressed in the urine of patients with a variety of solid tumours. Most studies have focused on evaluating the utility of UGP in the management of malignant gynaecological disease, although significant elevations have been observed in other types of malignancies. This study demonstrated that UGP is also overexpressed in a majority of Egyptian patients with advanced stage bladder cancer. The source of UGP in the urine of patients with malignant disease is the metabolic breakdown of hCG species, predominantly hCG beta subunit, originating in the tumour tissue. This is corroborated by previous reports that have demonstrated the presence of hCG beta subunit in the tissues and circulation of approximately 50% of patients with bladder cancer (Oliver *et al.*, 1988; Marcillac *et al.*, 1992). Other studies have shown that UGP was present in the urine of patients with hCG-producing bladder tumours (Iles *et al.*, 1990). Because UGP is the predominant hCG-derived species in urine, it is the most sensitive marker of hCG immunoreactivity for indicating the presence of malignancy. An additional factor contributing to the high level of UGP overexpression in this population of bladder cancer patients could be the relatively high proportion with advanced disease.

In this study population, UGP was demonstrated to be a sensitive and specific marker for malignancy. UGP was only marginally elevated in samples from normal individuals and in patients with benign urological disease. Mean UGP levels in patients with bladder cancer were 81-fold and 44-fold higher than those in normal individuals and patients with benign disease respectively. At the 95% and 100% specificity levels, overall sensitivities of 73% and 60%, respectively, were observed. A statistically significant increase in median UGP level as a function of stage and grade was observed, but no correlation with histological type, nodal involvement or bilharzial association was demonstrable.

The sensitivity of UGP for detecting malignancy in this population of Egyptian bladder cancer patients was comparable with or better than that of other tumour markers. At specificities of 95% and 100%, sensitivities of 73% and 60% respectively were observed. By comparison, at 95% specificity, urinary squamous cell antigen (SCC antigen), ferritin, CEA and TPA were elevated in 24%, 72%, 62% and 81% respectively, of patients with bladder cancer (El-Ahmady *et al.*, 1992*a*, *b*; Halim *et al.*, 1992). However, at 100% specificity, the sensitivities of ferritin, CEA and TPA dropped markedly to 34%, 23% and 34% respectively.

The UGP cut-offs used in this study are lower than those used in other studies reported in the literature. This could be due to several factors. First, the levels of UGP in cancer patients could be expected to vary according to tumour type. Second, the populations in this study were predominantly male. Earlier studies have shown that the normal range of UGP is measurably higher in post-menopausal women compared with males and premenopausal women (Lee *et al.*, 1991). Finally, this study used 24 urines for UGP determination and the majority of studies reported in the literature with this marker use spot urines, usually corrected for creatinine. Because spot or early-morning spot urines are more readily obtainable than timed 24 h urines, future studies will evaluate the correlation between 24 h urines and spot urines corrected for creatinine.

Owing to its high sensitivity for detecting individuals with malignant disease at a cut-off at which no false-positives were observed in patients with benign urological disease, the clinical value of UGP could be for the differential diagnosis of these patients, particularly in high-risk populations. The application of this marker for this use needs to be evaluated in further studies.

The use of UGP is facilitated by the fact that it is a highly stable marker that is measurable in urine, which is a readily obtained and non-invasive sample. Future studies will focus on evaluating UGP expression in early stage disease, as well as for monitoring and detecting recurrent disease.

**Acknowledgements**
The authors would like to thank the staff members of the Surgery Department, Egyptian National Cancer Institute, and the Department of Urology, Kasr El-Aini Hospital, Cairo University, for obtaining urine samples and providing clinical data for the patients with benign and malignant urinary tract disease.

### References

AL-SHUKRI S, ALWAN MH, NAYEF M AND RAHMAN AA. (1987). Bilharziasis in malignant tumours of the urinary bladder. *Br. J. Urol.*, **59**, 59-62.

ALFTHAN H, HAGLUND C, ROBERTS P AND STENMAN U-H. (1992). Elevation of free beta subunit of human choriogonadotropin and beta core fragment of human choriogonadotropin in the serum and urine of patients with malignant pancreatic and bilary disease. *Cancer Res.*, **52**, 4628-4633.

BEAHRS OH, HENSON DE, HUTTER RVP AND MYERS MH. (1988). *Manual for Staging of Cancer*, 3rd edn., pp. 193-195. JB Lippincott: Philadelphia.

BIRKEN S, ARMSTRONG EG, KOLKS MAG AND COLE LA. (1988). The structure of the human chorionic gonadotropin beta core fragment from pregnancy urine. *Endocrinology*, **123**, 572-583.

BLYTHE DL, WEHMANN RE AND NISULA BC. (1989). Carbohydrate composition of beta-core. *Endocrinology*, **125**, 2267-2272.

COLE LA. (1994). Beta-core fragment (beta-core, UGP or UGF). *Tumor Marker Update*, **6**, 69-75.

COLE LA AND NAM JH. (1989). Urinary gonadotropin fragment (UGF) measurements in the diagnosis and management of ovarian cancer. *Yale J. Biol. Med.*, **62**, 367-378.

DE MEDEIROS SF, AMATO F AND NORMAN RJ. (1991). Stability of immunoreactive beta-core fragment of hCG. *Obstet. Gynecol.*, **77**, 53-59.

EL-AHMADY O. (1988). Certain tumor markers in bilharzial patients in relation to bladder cancer. *J. Tumor Marker Oncol.*, **3**, 227-235.

EL-AHMADY O, HAMZA S, ABOUL-ELA M, HALIM A-B AND OEHR P. (1990). The value of tissue polypeptide antigen in Egyptian bladder cancer patients. In *Recent Results in Tumor Diagnosis and Therapy*, Klapdor, R. (ed.) pp. 230-236. W. Zuckschwerdt: Munich.

EL-AHMADY O, HALIM A-B, GAD EL MAWALA N AND MOHAMADIN A. (1991*a*). Serum and urine immunoglobulins in Egyptian bladder cancer patients. *Egypt. J. Tumor Marker Oncol.*, **2**, 91-97.

EL-AHMADY O, BARAKAT M, EL-GHAZAWY IMH AND AFIFY M. (1991*b*). The clinical value of squamous cell carcinoma antigen and tumor-associate trypsin inhibitor in bladder cancer. *Egypt. J. Tumor Marker Oncol.*, **2**, 79-82.

EL-AHMADY O, HAMZA S, ABOUL-ELA M, HALIM A-B AND OEHR P. (1992*a*). Serum and urine ferritin in Egyptian bladder cancer patients. *J. Tumor Marker Oncol.*, **7**, 69-89.

EL-AHMADY O, HAMZA S, ABOUL-ELA M, HALIM A-B AND OEHR P. (1992*b*). Urinary squamous cell carcinoma antigen in Egyptian bladder cancer patients. *Egypt J. Tumor Marker Oncol.*, **3**, 35-41.

EL-SEBAI I. (1961). Cancer of the bladder in Egypt. *Kasr El-Aini J. Surg.*, **2**, 182-241.

ENDO TR, NISHIMUR R, SAITO S AND KANAZAWA K. (1992). Carbohydrate structures of beta-core fragment of human chorionic gonadotropin isolated from a pregnant individual. *Endocrinology*, **130**, 2052-2058.

FRANCHIMONT P, GASPARD U, REUTER A AND HEYNEN G. (1972). Polymorphism of protein and polypeptide hormones. *Clin. Endocrinol.*, **1**, 315-336.

HALIM A-B, EL-AHMADY O, HAMZA S, ABOUL-ELA M AND OEHR P. (1992). Simultaneous determination of urinary CEA, ferritin, and TPA in Egyptian bladder cancer patients. *Int. J. Biol. Markers*, **7**, 234-239.

HALIM A-B, EL-AHMADY O, HAMZA S, ABOUL-ELA M AND OEHR P. (1993). Serum TPS versus TPA in Egyptian bladder cancer patients. *Int. J. Biol. Markers*, **8**, 221-226.

HALIM A-B, BARAKAT M, EL-ZAYAT AM, DAW M AND EL-AHMADY O. (1994). Urinary beta-hCG in benign and malignant urinary tract diseases. *Disease Markers*, **12**, 109-115.

ILES RK, LEE CL, OLIVER TD AND CHARD T. (1990). Composition of intact hormone and free subunits in the human chorionic gonadotropin-like material found in serum and urine of patients with carcinoma of the bladder. *Clin. Endocrinol.*, **32**, 355-364.

KARDANA A, TAYLOR ME, SOUTHALL PJ AND BOXER GM. (1988). Urinary gonadotropin peptide-isolation and purification, and its immunohistochemical distribution in normal and neoplastic tissues. *Br. J. Cancer*, **58**, 281-286.

KATO Y AND BRAUNSTEIN GD. (1988). Beta-core fragment is a major form of immunoreactive urinary chorionic gonadotropin in human pregnancy. *J. Clin. Endocrinol. Metab.*, **66**, 1197-1201.

KHALED HM. (1993). Bladder cancer and bilharziasis today. *Cancer J.*, **6**, 65-71.

LEE CL, ILES RK, SHEPHERD JH, HUDSON CN AND CHARD T. (1991). The purification and development of a radioimmunoassay for beta-core fragment of human chorionic gonadotropin in urine: application as a marker of gynecological cancer in premenopausal and postmenopausal women. *J. Endocrinol.*, **130**, 481-489.

MCGILL J, COLE LA, NAM JH AND THORSON A. (1990). Urinary gonadotropin fragment (UGF): a potential 'marker' of colorectal cancer. *J. Tumor Marker Oncol.*, **5**, 175-177.

MARCILLAC I, TROALEN F, BIDART J, GHILLANI P, RIBRAG V, ESCUDIER B, MALASSAGNE B, DROZ J, LHOMME C, ROUGIER P, DUVILLARD P, PRADE M, LUGAGNE P, RICHARD F, POYNARD T, BOUHUON C, WANDS J AND BELLET D. (1992). Free human chorionic gonadotropin beta subunit in gonadal and nongonadal neoplasms. *Cancer Res.*, **52**, 3901-3907.

MUSTACCHI P AND SHIMKIN MB. (1958). Cancer of the bladder and infestation with *Schistosoma Haematobium*. *J. Natl. Cancer Inst.*, **20**, 825-842.

NAM JH, CHAMBERS JT, SCHWARTZ PE AND COLE LA. (1990*a*). Urinary gonadotropin fragment, a new tumor marker: IV. Use in endometrial cancers and uterine mixed mullerian tumors. *Gynecol. Oncol.*, **39**, 352–357.

NAM JH, CHANG KC, CHAMBERS JT AND SCHWARTZ PE. (1990*b*). Urinary gonadotropin fragment, a new tumor marker. III. Use in cervical and vulvar cancers. *Gynecol. Oncol.*, **38**, 66–70.

NORMAN RJ, BUCK RH, AKAR B AND MAYET N. (1990). Detection of a small molecular species of human chorionic gonadotropin in the urine of patients with carcinoma of the cervix and cervical intraepithelial neoplasia: comparison with other assays for human chorionic gonadotropin and its fragments. *Gynecol. Oncol.*, **37**, 254–259.

OLIVER RTD, STEPHENSON C, COLLINO CE AND PARKINSON MC. (1988). Clinicopathological significance of immunoreactive beta-hCG production by bladder cancer. *Mol. Biother.*, **1**, 43–45.

PAPAPETROU PD, SAKARELOU NP, BRAOUZI H AND FESSAS PH. (1980). Ectopic production of human chorionic gonadotropin (hCG) in the urine as a screening procedure. *Cancer*, **45**, 2583–2592.

YOSHIMURA M, NISHIMURA R, MUROTANI A, MIYAMOTO Y, NAKAGAWA T, HASEGAWA K, KOIZUMI T, SHII K, BABA S AND TSUBOTA N. (1994). Assessment of urinary beta-core fragment of human chorionic gonadotropin as a new tumor marker of lung cancer. *Cancer*, **73**, 2745–2752.